# Prevalence and Determinants of Abnormal Ambulatory Blood Pressure and Circadian Patterns Among Adults in Ido Ekiti, Nigeria: A Community‐Based Study

**DOI:** 10.1155/ijhy/6016519

**Published:** 2026-06-29

**Authors:** Ebenezer Adekunle Ajayi, Okechukwu S. Ogah, Joseph Olusesan Fadare

**Affiliations:** ^1^ Department of Medicine, Federal Teaching Hospital, Ido Ekiti, Nigeria, fethahrm.com; ^2^ Department of Medicine, Ekiti State University, Ado Ekiti, Nigeria, eksu.edu.ng; ^3^ Department of Medicine, University of Ibadan, Ibadan, Nigeria, ui.edu.ng

**Keywords:** ambulatory blood pressure monitoring, circadian rhythm, hypertension, Nigeria, nondipping, obesity, socioeconomic status

## Abstract

**Background:**

Hypertension is the leading cause of global cardiovascular diseases and deaths, but its management remains poor in sub‐Saharan Africa. Ambulatory blood pressure monitoring (ABPM) provides more detailed information on variations in blood pressure during the day and circadian patterns than office measurements. Unfortunately, there is a paucity of information regarding ABPM patterns among Nigerian adults.

**Objective:**

To determine the prevalence and circadian patterns of abnormal ambulatory blood pressure, as well as to identify sociodemographic, anthropometric and lifestyle predictors of systolic and diastolic dipping status in the adult population of Ido Ekiti, Nigeria.

**Methods:**

A community‐based cross‐sectional study involving 352 adults aged ≥ 18 years was conducted using a multistage sampling method. Office blood pressure and 24‐h ABPM were recorded with validated automated devices. Dipping patterns were classified as dipper (10%–20%), nondipper (< 10%), reverse dipper (no decrease or increase) or extreme dipper (≥ 20%). Logistic regression was used to identify predictors of nondipping patterns.

**Results:**

The mean age was 48.6 ± 17.9 years, and 55.7% of the participants were females. Thirty‐nine‐point‐eight percentage of the subjects had abnormal 24‐h blood pressure (≥ 130/80 mmHg). Only 22.2% and 36.9% showed normal systolic and diastolic dipping, respectively. Nondipping was associated with higher income (₦70,000–₦500,000; adjusted odds ratio [AOR] 7.91–104.0; *p* < 0.05) and central obesity (AOR 1.67 × 10^3^–1.95 × 10^4^; *p* < 0.05). Elevated 24‐h systolic blood pressure increased the likelihood of nondipping (AOR 6.35–7.63), whereas abnormal diastolic blood pressure appeared to have a protective effect (AOR 0.09–0.84). Following WHO physical activity recommendations resulted in a reduction of systolic nondipping (AOR 0.23; *p* = 0.010), whereas it was associated with an increase in diastolic nondipping (AOR 3.97; *p* = 0.004). Being male and having a higher office diastolic blood pressure level were protective against systolic nondipping.

**Conclusion:**

Abnormal circadian blood pressure patterns are frequent among Nigerian adults, and nondipping is the predominant pattern. Socioeconomic status, central adiposity and 24‐h BP load are the most influential factors. The promotion of ABPM in primary care is a vital step in detecting hypertension early and managing it in a personalised way.

## 1. Introduction

Hypertension remains one of the most common noncommunicable diseases worldwide and a significant factor in cardiovascular morbidity and mortality. It significantly contributes to strokes, heart failure, kidney issues and premature death [1, 2]. Despite advances in diagnosis and treatment, hypertension management worldwide remains inadequate, particularly in low‐ and middle‐income countries where awareness, treatment and control are substantially low [3, 4]. In Nigeria and other sub‐Saharan African nations, the burden of hypertension continues to rise, driven by urbanisation, dietary changes, physical inactivity and an ageing population [1, 5].

Accurate diagnosis is essential for effective management. However, office blood pressure (BP) measurement can be affected by factors such as anxiety, physical activity and the white‐coat effect, leading to potential misclassification, while masked hypertension often remains undetected. Both conditions pose cardiovascular risks similar to those of sustained hypertension [6]. Recent guidelines from the World Health Organization [7] and the International Society of Hypertension [8] emphasise the limitations of relying solely on clinic measurements and advise confirming elevated office BP with out‐of‐office methods. Ambulatory blood pressure monitoring (ABPM) is recommended as the preferred method for this purpose, particularly in diagnosing white‐coat and masked hypertension, evaluating nocturnal BP, detecting abnormal circadian patterns and BP variability that offer prognostic information beyond what office readings can provide and is considered the gold standard for risk stratification and therapeutic decisions [9].

Nocturnal hypertension and abnormal dipping patterns are strongly associated with target‐organ damage, including microalbuminuria, cerebrovascular disease, left ventricular hypertrophy and increased mortality [7, 10]. However, despite these risks and other benefits, the use of ABPM in Nigeria and many parts of sub‐Saharan Africa remains limited due to high costs, limited access and low awareness among healthcare providers [11–13]. The few Nigerian studies, mostly hospital‐based and involving small or selective samples, or lacking comprehensive analyses of circadian rhythms [1, 14–17], leave a significant gap in community‐based data on ambulatory BP patterns, nocturnal BP behaviour and their determinants.

This study addresses this gap by conducting a community‐based cross‐sectional evaluation of 24‐h ambulatory BP among adults in Ido Ekiti, utilising validated ABPM devices and standardised criteria for dipping status. We determined the prevalence of abnormal ambulatory BP patterns and explored sociodemographic, anthropometric and lifestyle factors linked to systolic and diastolic dipping behaviour. We hypothesised that nondipping and nocturnal hypertension would be common in this population and independently associated with older age, excess adiposity and lower physical activity levels. The findings from this study are expected to offer crucial baseline data to inform clinical practice, support the adoption of standard hypertension guidelines in resource‐constrained settings and influence public health strategies for hypertension management in Nigeria.

## 2. Methods

### 2.1. Study Design and Setting

A community‐based cross‐sectional study was performed among apparently healthy adults in Ido Ekiti, Ekiti State, Nigeria. Ido Ekiti is a semiurban district that combines socioeconomic and demographic characteristics representative of much of south‐western Nigeria. Data collection occurred from June to October 2025.

### 2.2. Study Population and Sampling

The study population consisted of adults aged 18 years and older, residents of the study area for at least 6 months and who had given their consent. The exclusion criteria were individuals with arrhythmias that may cause ABPM inaccuracies, those with upper‐arm deformities that make cuff placement impossible, pregnant women and persons with incomplete ABPM recordings.

A multistage sampling technique was utilised to recruit study participants and improve the sample’s representativeness. Initially, quarters within Ido Ekiti were selected through simple random sampling by balloting, based on a comprehensive list of recognised quarters obtained from community administrative records. Subsequently, these quarters were mapped, and households within each quarter were chosen using systematic random sampling. The sampling interval was calculated based on the estimated number of households in each selected quarter and the proportionate allocation of sample sizes. Finally, eligible adults residing in the selected households were identified, and when more than one eligible individual was present, a single participant was randomly selected. The sampling frames employed at the various stages of participant selection consisted of: (i) a comprehensive list of recognised quarters in Ido Ekiti obtained from community administrative records, serving as the sampling frame for quarter selection; (ii) a listing of households within the selected quarters, serving as the sampling frame for household selection; and (iii) all eligible adults aged 18 years and above residing in the selected households at the time of the survey, which constituted the sampling frame for participant selection.

The sample size was calculated to detect a hypertension prevalence of 28.9% [18] with a precision of 5% and a 95% confidence level. Therefore, the minimum number of participants required was 316.

### 2.3. Data Collection and Measurements

Data on sociodemographic factors, lifestyle‐related factors and dietary history of respondents were obtained using a standardised questionnaire derived from a modified WHO STEPS Instrument for noncommunicable diseases [19]. The anthropometric measurements of the respondents, including weight, height and waist circumference, were taken according to the standard WHO protocols. Body mass index (BMI) was calculated as weight/height [2] (kg/m^2^) and categorised according to WHO cut‐offs.

### 2.4. ABPM

Ambulatory BP was obtained using validated oscillometric ABPM monitors (CONTEC ABPM60®, China). The instruments were programmed to take measurements every half an hour during the day (07:00–22:00) and every 1 hour at night (22:00–07:00). Individuals were allowed to carry on with their everyday activities; however, they were discouraged from engaging in vigorous exercises and were required to note their sleep and wake times. A valid ABPM recording was defined as at least 70% of readings being valid, with at least one valid reading per hour.

### 2.5. Operational Definitions

• 24‐h hypertension: mean 24‐h BP ≥ 130/80 mmHg.
• Daytime hypertension: mean daytime BP ≥ 135/85 mmHg.
• Night‐time hypertension: mean nocturnal BP ≥ 120/70 mmHg.
• High ambulatory BP variability: 24‐h systolic BPV > 10% using the coefficient of variation.
• Abnormal BP load: > 25% of systolic/diastolic readings ≥ 135/85 and ≥ 120/70 mm Hg during day and night, respectively.
• Dipping pattern: these were determined by the percentage decrease of the mean nocturnal systolic and diastolic BP in relation to the daytime values, with dippers defined as a 10%–20% reduction; nondippers as less than a 10% reduction; extreme dippers as more than a 20% reduction; and reverse dippers as having BP at night higher than during the day.

All definitions were in line with the existing ESH/ISH guidelines [20].

### 2.6. Statistical Analysis

SPSS Version 26 (IBM Corp., Armonk, NY, USA) was used for data analysis. Continuous variables were presented as means ± SD, and categorical variables as counts and percentages. Appropriate statistical tests were applied, including an independent‐samples *t*‐test, a Mann–Whitney *U* test and a *χ* ^2^ test. Binary logistic regression models were used to identify factors determining dipping (dipper and extreme dipper) and nondipping (nondipper and reverse dipper) for both systolic and diastolic BP. Multivariate analyses were also performed. Initially, Pearson correlation coefficients (*r*) and independent‐samples *t*‐tests were used to assess crude relationships between ambulatory systolic/diastolic BP and variables such as age, sex, BMI, smoking, alcohol intake and physical activity. Variables showing statistical significance (*p* < 0.10) in bivariate analysis were included in multivariable linear regression models to identify independent predictors of mean 24‐h systolic and diastolic BP. Multicollinearity was assessed using variance inflation factors (VIF < 5). Results included regression coefficients (*β*) with 95% confidence intervals (CIs) and standardised beta values to indicate the strength and direction of associations. Separate models were developed for systolic and diastolic BP, and logistic regression analyses identified factors associated with ambulatory hypertension after adjustment for key confounders. Variables with *p* < 0.20 in bivariate analyses, together with clinically relevant covariates, were considered for inclusion in multivariable models. Adjusted odds ratios (aOR) with 95% CIs were reported. Statistical significance was set at *p* < 0.05.

### 2.7. Ethical Considerations

The Research and Ethics Committee of the Federal Teaching Hospital, Ido Ekiti, approved the research (Protocol No: ERC/2025/05/06/1244A). Voluntary written consent was obtained from all participants. Confidentiality was ensured through data anonymisation, and all activities were in accordance with the Declaration of Helsinki (2013 revision).

### 2.8. Quality Control

The field workers (consisting of four medical doctors, six medical laboratory scientists/technicians and four research assistants) underwent a standard 1‐week training programme covering questionnaire administration, anthropometric measurements and proper handling of the ABPM device. All the measuring instruments were calibrated before and during the study. Unannounced supervisory visits were the means through which the protocol implementation was checked, and 10% of the questionnaires were randomly selected for review to verify their accuracy and completeness.

## 3. Results

### 3.1. Sociodemographic, Lifestyle and Dietary Characteristics of the Participants

The research sample included 352 adults. Their mean age was 48.6 ± 17.9 years (95% CI: 46.7–50.5), with the 40–59‐year age group being the most common (41.8%). Females made up 55.7% of the total sample, and most participants were Yoruba (89.2%), married (59.1%) and had a tertiary education (65.1%). Over half of the participants (52.0%) reported monthly incomes between ₦70,000 and ₦300,000 ($52–$220), as shown in Table 1. The mean BMI was 23.8 ± 4.4 kg/m^2^, with 58.8% of the subjects of normal weight, 17.9% overweight and 12.2% obese based on this measurement. The mean waist‐to‐hip ratio was 0.90 ± 0.07 (95% CI: 0.89–0.91). Nearly all participants were nonsmokers (99.4%), and 64.8% had consumed alcoholic beverages in the past year. The majority were physically active, with 78.6% meeting the WHO criterion of ≥ 600 MET‐minutes per week.

**TABLE 1:** Sociodemographic, lifestyle and dietary characteristics of participants (*n* = 352).

Variable	Category	Frequency (*n*)	Percentage (%)	95% confidence interval
Age group	18–39	107	30.4	25.6–35.2
	40–59	147	41.8	36.7–46.9
	≥ 60	98	27.8	23.1–32.5

Gender	Male	156	44.3	39.1–49.5
	Female	196	55.7	50.5–60.9

Monthly income (₦)	< 70,000	96	27.3	22.6–32.0
	70,000–300,000	183	52	46.7–57.3
	301,000–500,000	26	7.4	4.6–10.2
	> 500,000	25	7.1	4.3–9.9

Educational level	Tertiary	229	65.1	60.0–70.2
	Secondary	81	23	18.5–27.5
	Primary	37	10.5	7.3–13.7
	No formal education	2	0.6	0.0–1.4

Current tobacco use	Yes	2	0.6	0.0–1.4
	No	350	99.4	98.6–100.0

Consumed alcohol in past 12 months	Yes	228	64.8	59.8–69.8
	No	124	35.2	30.2–40.2

Access to healthcare	Public facility	304	86.4	82.8–90.0
	Private facility	30	8.5	5.6–11.4

Meets WHO physical activity recommendations	Yes (≥ 600 MET‐min/week)	277	78.6	74.4–83.0
	No (< 600 MET‐min/week)	75	21.4	17.0–25.6

Body mass index (BMI)	—	—	—	23.8 ± 4.4 (23.3–24.3)

BMI category	Normal (< 25)	207	58.8	53.7–63.9
	Overweight (25–29.9)	63	17.9	13.9–21.9
	Obese (≥ 30)	43	12.2	8.8–15.6

Waist‐to‐hip ratio (WHR)	—	—	—	0.90 ± 0.07 (0.89–0.91)

*Note:* Data are presented as frequency (percentages) along with 95% confidence intervals. Continuous variables are expressed as mean ± standard deviation (SD) with 95% CI.

The average household size was 4.9 ± 1.4. The use of salt in cooking was very common, as 44.1% always and 27.4% often adding salt. Although 72.3% confirmed that they seldom or never ate processed salty foods, 21.7% admitted to consuming them occasionally. Most of the respondents (62.5%) perceived their salt intake as ‘just right’, while 33.2% thought it was ‘too little’.

### 3.2. Mean Hourly Ambulatory BP Trend of the Study Population

**FIGURE 1:**
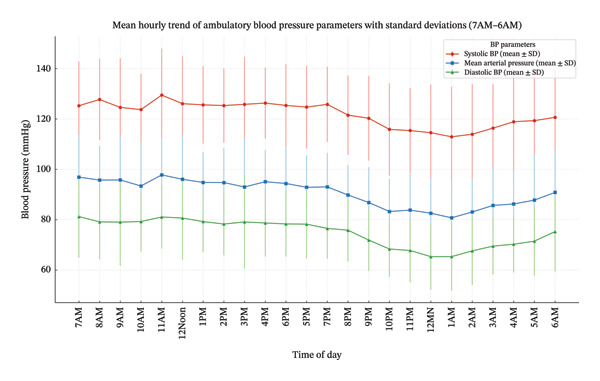
Mean hourly trend of ambulatory blood pressure with standard deviations (7:00 am–6:00 am). Note: This figure shows the mean ± standard deviation of systolic blood pressure (SBP), mean arterial pressure (MAP) and diastolic blood pressure (DBP) recorded hourly over a 24‐h span.

The diurnal pattern is illustrated in Figure 1, a descriptive analysis of the aggregate participants’ fixed clock intervals, showing that BP rises from 7:00 a.m. to 9:00 a.m. and remains relatively stable for the rest of the day. In the evening, BP gradually decreases, reaching its lowest value between 11:00 p.m. and 3:00 a.m. A rise occurs again after 4:00 a.m. The standard deviation bars indicate greater variation among individuals during the day than at night, which may be due to differences in responses to daily activities.

### 3.3. Comparative Distribution of Ambulatory BP by Age and Gender

**FIGURE 2:**
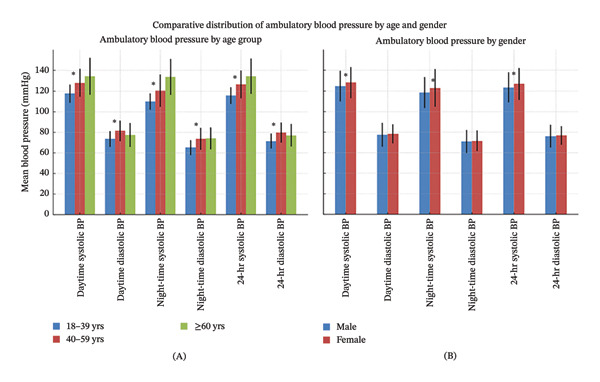
Comparative distribution of ambulatory blood pressure by age and gender. Note: Panel A displays the mean daytime, night‐time and 24‐h systolic and diastolic blood pressure values across three age groups (18–39 years, 40–59 years and ≥ 60 years). Mean ambulatory blood pressure increases progressively with age, with older participants exhibiting higher systolic and diastolic pressures during both daytime and night‐time. Panel B compares mean ambulatory blood pressure between males and females, indicating slightly higher systolic and diastolic blood pressure values in males across all measurement periods. Error bars represent standard deviations. ^∗^ *p* < 0.05.

Most age‐related BP parameters showed significant differences (*p* < 0.001) (Table 2 and Figure 2). Mean office systolic BP increased from 121.2 ± 16.3 mmHg (18–39 years) to 149.5 ± 27.7 mmHg (≥ 60 years). Similar changes in BP values were also observed across day, night and 24‐h periods. Older adults also exhibited higher pulse pressures and BP loads, particularly nocturnal systolic load, which rose from 15.7 ± 16.2% to 54.4 ± 31.6% across age categories.

**TABLE 2:** Comparison of ambulatory blood pressure parameters by gender.

Variable	Male (mean ± SD)	Female (mean ± SD)	Total (mean ± SD)	*F* value	*p* value
Office systolic BP (mmHg)	133.1 ± 20.5	136.1 ± 26.4	134.8 ± 24.0	1.33	0.249
Office diastolic BP (mmHg)	83.2 ± 13.6	82.9 ± 16.4	83.0 ± 15.2	0.03	0.858
Office heart rate (bpm)	74.7 ± 11.1	82.8 ± 14.3	79.2 ± 13.6	32.25	< 0.001
Daytime systolic BP (mmHg)	124.6 ± 15.0	128.0 ± 15.2	126.5 ± 15.2	4.44	0.036
Daytime diastolic BP (mmHg)	77.2 ± 11.5	78.3 ± 9.3	77.8 ± 10.4	0.84	0.36
Daytime pulse pressure (mmHg)	47.4 ± 8.5	50.1 ± 10.9	48.9 ± 10.0	6.7	0.01
Daytime pulse rate (bpm)	75.4 ± 7.4	79.8 ± 9.4	77.8 ± 8.8	21.74	< 0.001
Night‐time systolic BP (mmHg)	118.3 ± 14.8	122.9 ± 18.2	120.8 ± 16.9	6.43	0.012
Night‐time diastolic BP (mmHg)	70.8 ± 11.0	71.5 ± 9.9	71.2 ± 10.4	0.3	0.583
Night‐time pulse pressure (mmHg)	47.4 ± 8.3	51.4 ± 12.0	49.7 ± 10.7	12.26	0.001
Night‐time pulse rate (bpm)	67.8 ± 6.5	71.6 ± 9.3	69.8 ± 8.3	18.31	< 0.001
24‐h systolic BP (mmHg)	123.3 ± 14.5	126.7 ± 15.5	125.2 ± 15.2	4.43	0.036
24‐h diastolic BP (mmHg)	76.1 ± 11.1	76.6 ± 8.9	76.4 ± 9.9	0.25	0.619
24‐h pulse pressure (mmHg)	47.2 ± 7.6	50.1 ± 10.2	48.8 ± 9.2	8.61	0.004
24‐h pulse rate (bpm)	73.8 ± 6.8	77.7 ± 9.1	75.9 ± 8.4	19.46	< 0.001
Daytime systolic BP Load (%)	26.3 ± 29.3	33.3 ± 30.1	30.2 ± 29.9	4.84	0.029
Night‐time systolic BP Load (%)	37.9 ± 37.5	47.3 ± 35.7	43.1 ± 36.7	5.71	0.017
24‐h systolic BP Load (%)	31.6 ± 30.3	39.7 ± 31.5	36.1 ± 31.2	5.98	0.015
% drop in systolic BP	4.9 ± 7.2	4.3 ± 7.2	4.5 ± 7.2	0.64	0.423
24‐h SBP variability (mmHg)	11.6 ± 3.6	13.3 ± 4.8	12.6 ± 4.4	14.55	< 0.001
24‐h DBP variability (mmHg)	16.8 ± 5.6	18.6 ± 7.2	18.0 ± 6.4	18.83	< 0.001

*Note: F* values are from one‐way ANOVA comparing males and females.

Abbreviations: BP, blood pressure; bpm, beats per minute; DBP, diastolic blood pressure; SBP, systolic blood pressure; SD, standard deviation.

Males and females had comparable mean office BP values (*p* > 0.05), but females exhibited significantly higher night‐time systolic BP (122.9 ± 18.2 vs. 118.3 ± 14.8 mmHg, *p* = 0.012) and increased nocturnal pulse pressure and BP variability. Males, however, showed lower mean heart rates during both day and night (*p* < 0.001).

### 3.4. Predictors of 24‐Hour BP

**FIGURE 3:**
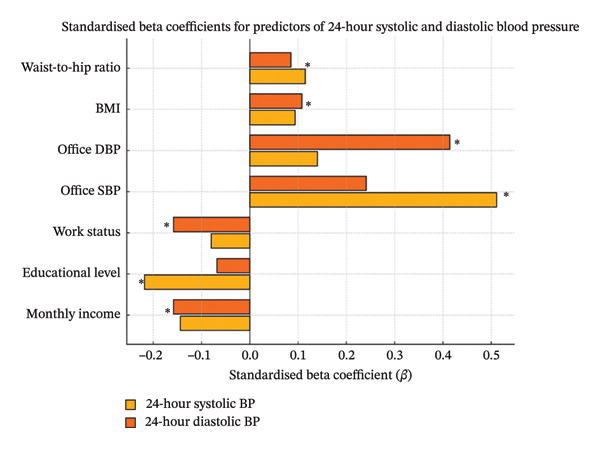
Standardised beta coefficients for predictors of 24‐h systolic and diastolic blood pressure. Horizontal bars represent standardised beta coefficients derived from multiple linear regression analysis. Bars to the right of the zero‐reference line indicate positive associations with ambulatory blood pressure; bars to the left indicate inverse associations. ^∗^Statistically significant predictors. Note: Model summary: 24‐h systolic BP: *R* = 0.790, *R* ^2^ = 0.624, adjusted *R* ^2^ = 0.602, *F* (13, 225) = 28.71, *p* < 0.001. 24‐h diastolic BP: *R* = 0.661, *R* ^2^ = 0.437, Adjusted *R* ^2^ = 0.404, *F* (13, 225) = 13.42, *p* < 0.001.

Both the 24‐h systolic and diastolic BP multiple linear regression models were statistically significant (Systolic: *R* ^2^ = 0.624, *F* (13, 225) = 28.71, *p* < 0.001; diastolic: *R* ^2^ = 0.437, *F* (13, 225) = 13.42, *p* < 0.001), thus accounting for 62% and 44% of the variances in ambulatory systolic and diastolic pressures, respectively (Figure 3). In both models, clinic BP readings emerged as the strongest predictors, highlighting their close correlation with ambulatory BP readings. Office systolic BP had the most significant influence on 24‐h systolic load (*β* = 0.511, *p* < 0.001), while office diastolic BP was the main determinant of 24‐h diastolic pressure (*β* = 0.414, *p* < 0.001). Socioeconomic factors, such as education level and monthly income, were negatively correlated with both, indicating that improved socioeconomic status leads to lower BP over time. Indices of adiposity, including BMI and waist–hip ratio, had modest but positive associations with both systolic and diastolic pressures.

### 3.5. Distribution of Abnormal Ambulatory BP Patterns Among Adult Participants

Abnormal ambulatory BP readings were common (Table 3). Elevated daytime systolic BP was observed in 26.7% of participants and elevated diastolic BP in 25.6%. Nocturnal abnormalities were more significant, with elevated systolic and diastolic BPs in 44.0% and 50.3% of cases, respectively. Overall, 39.8% had abnormal 24‐h mean BP (≥ 130/80 mmHg). BP load abnormalities were also frequent: 39.5% (daytime systolic), 43.5% (daytime diastolic), 56.0% (nocturnal systolic) and 65.9% (nocturnal diastolic). Similarly, 50.3% and 56.8% showed elevated 24‐h systolic and diastolic BP loads, respectively.

**TABLE 3:** Distribution of ambulatory blood pressure parameters among adult participants (*N* = 352).

Parameter	Category	Frequency (*n*)	Percentage (%)	95% confidence interval
Daytime mean systolic BP	Elevated	94	26.7	22.1–31.3
	Normal	258	73.3	68.7–77.9

Daytime mean diastolic BP	Elevated	90	25.6	21.0–30.1
	Normal	262	74.4	69.9–79.0

Night‐time mean systolic BP	Elevated	155	44.0	38.8–49.2
	Normal	197	56.0	50.8–61.2

Night‐time mean diastolic BP	Elevated	177	50.3	45.1–55.5
	Normal	175	49.7	44.5–54.9

24‐h mean systolic BP	Elevated	111	31.5	26.7–36.4
	Normal	241	68.5	63.6–73.3

24‐h mean diastolic BP	Elevated	114	32.4	27.5–37.3
	Normal	238	67.6	62.7–72.5

24‐h mean BP (≥ 130/80 mmHg)	Elevated	140	39.8	34.7–44.9
	Normal	212	60.2	55.1–65.3

Daytime systolic BP load	Abnormal	139	39.5	34.4–44.6
	Normal	213	60.5	55.4–65.6

Daytime diastolic BP load	Abnormal	153	43.5	38.3–48.6
	Normal	199	56.5	51.4–61.7

Night‐time systolic BP load	Abnormal	197	56.0	50.8–61.2
	Normal	155	44.0	38.8–49.2

Night‐time diastolic BP load	Abnormal	232	65.9	61.0–70.9
	Normal	120	34.1	29.1–39.0

24‐h systolic BP load	Abnormal	177	50.3	45.1–55.5
	Normal	175	49.7	44.5–54.9

24‐h diastolic BP load	Abnormal	200	56.8	51.6–62.0
	Normal	152	43.2	38.0–48.4

BP variability (24‐h systolic)	High	241	68.5	63.6–73.3
	Normal	111	31.5	26.7–36.4

BP variability (24‐h diastolic)	High	335	95.2	92.9–97.4
	Normal	17	4.8	2.6–7.1

*Note:* Data are presented as frequency (*n*) and percentage (%). 95% confidence intervals were calculated using the binomial approximation method.

Abbreviation: BP, blood pressure.

### 3.6. Predictors of Abnormal 24‐Hour Systolic BP (≥ 130 mmHg) and Diastolic BP (≥ 80 mmHg) Among the Participants

As mentioned above, a logistic regression analysis was performed to evaluate predictors of abnormal 24‐h systolic BP. Older age (OR = 0.687, 95% CI: 0.513–0.921, *p* = 0.012) and higher office systolic BP (OR = 0.870, 95% CI: 0.807–0.938, *p* < 0.001) were independently associated with lower odds of abnormal 24‐h systolic BP, consistent with a white‐coat hypertension effect. Physical activity showed a small but significant positive association (OR = 1.004, 95% CI: 1.000–1.007, *p* = 0.038). Obesity (OR = 4929.75; *p* < 0.001) and overweight (OR = 5136.77; *p* = 0.001) were strong predictors of abnormal diastolic BP. Higher education and the male gender were protective.

**FIGURE 4:**
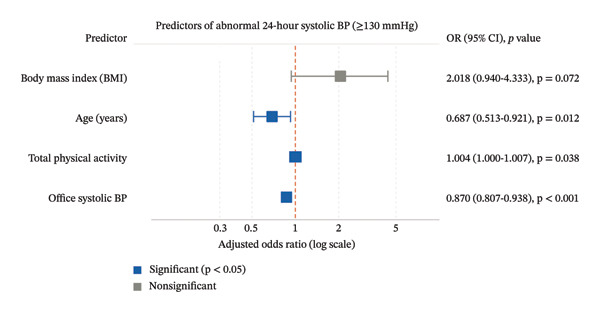
Predictors of abnormal 24‐h systolic blood pressure (≥ 130 mmHg) among the participants. Note: The forest plot displays adjusted odds ratios (ORs) and 95% confidence intervals (CIs) derived from a multivariable logistic regression model that identifies predictors of abnormal 24‐h systolic blood pressure (≥ 130 mmHg). The vertical red dashed line indicates the null value (OR = 1.0), signifying no association. Variables with 95% CIs that do not cross this line are deemed statistically significant predictors at *p* < 0.05.

**FIGURE 5:**
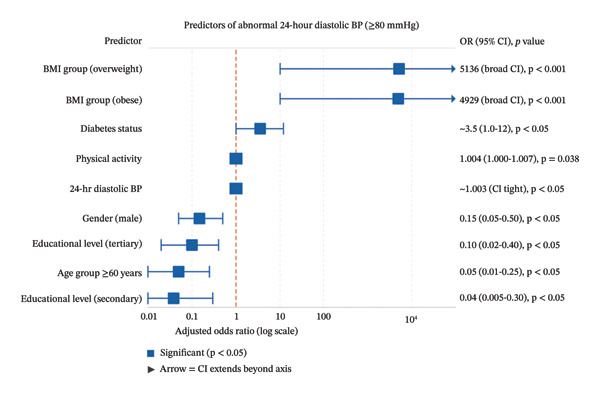
Predictors of abnormal 24‐h diastolic blood pressure (≥ 80 mmHg) among the participants. Note: The forest plot displays adjusted odds ratios (ORs) and 95% confidence intervals (CIs) from a multivariable logistic regression model that identifies independent predictors of abnormal 24‐h diastolic blood pressure (defined as ≥ 80 mmHg). The red dashed vertical line indicates the null value (OR = 1.0), signifying no association. Predictors with CIs that do not cross the null line are statistically significant at *p* < 0.05. The model included sociodemographic, behavioural and clinical variables, with 231 valid cases analysed.

Figure 4 presents a forest plot of the regression model for systolic BP (*F* (11, 269) = 12.62, *p* < 0.001), which explains 34.0% of the variance with age, education, work status and physical activity as the main predictors. The diastolic model (*F* (11, 269) = 3.73, *p* < 0.001), also illustrated in Figure 5, explains 13.2% of the variance, with work status, total weekly activity and average daily activity as significant predictors.

### 3.7. Circadian Ambulatory BP Profiles

**FIGURE 6:**
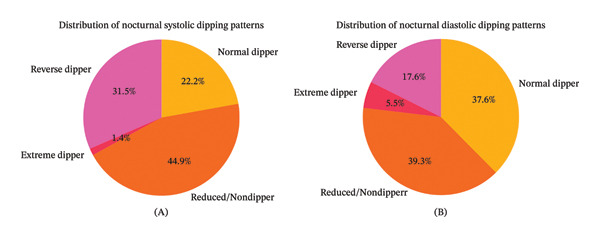
Distribution of nocturnal systolic and diastolic dipping patterns among participants. Note: Panel A shows the proportions of nocturnal systolic dipping categories, while Panel B displays diastolic dipping patterns. Dipping status was classified based on the percentage decline in mean nocturnal blood pressure relative to daytime mean blood pressure. Normal dipping was defined as a 10%–20% decline, reduced/nondipping as < 10%, extreme dipping as > 20%, and reverse dipping as an increase in nocturnal blood pressure compared with daytime blood pressure.

Nocturnal dipping abnormalities were common (Figure 6). For systolic BP, only 22.2% exhibited normal dipping, while 49.9% were reduced or nondippers and 31.5% were reverse dippers. For diastolic BP, 36.9% were normal dippers, 38.6% nondippers and 17.3% reverse dippers. High 24‐h BP variability was observed in 68.5% for systolic and 95.2% for diastolic pressures.

Binary logistic regression analyses were conducted to identify the factors influencing systolic and diastolic BP dipping patterns (Tables 4 and 5). Both models were significantly different from null models (systolic: *χ* ^2^ (22) = 102.02, *p* < 0.001; diastolic: *χ* ^2^ (22) = 96.27, *p* < 0.001), accounting for 49.2% and 43.6% of the variance in dipping status, respectively. The systolic model had a good fit (Hosmer–Lemeshow *χ* ^2^ = 14.81, *p* = 0.063) with high predictive performance (86.4%), whereas the diastolic model had fair accuracy (72.0%) and a modest fit (Hosmer–Lemeshow *χ* ^2^ = 14.81, *p* = 0.008).

**TABLE 4:** Binary logistic regression showing predictors of systolic BP nondipping among the participants.

Predictor	AOR (exp *B*)	95% CI	*p* value	Direction
Male gender	0.4	0.17–0.98	0.044	Protective
Income ₦70,000–₦300,000	104	8.46–1279	< 0.001	Increased risk
Income ₦301,000–₦500,000	96.5	10.3–902	< 0.001	Increased risk
Meets WHO METS (≥ 600 METS)	0.23	0.08–0.70	0.01	Protective
Elevated office DBP	0.15	0.03–0.66	0.013	Protective
Waist‐to‐hip ratio	1.95 × 10^4^	9.6–3.96 × 10^7^	0.011	Increased risk
Abnormal 24‐h SBP (≥ 130 mmHg)	7.63	1.29–45.03	0.025	Increased risk
24‐h DBP (continuous)	0.84	0.75–0.94	0.003	Protective

*Note:* *p* values < 0.05 indicate statistical significance.

Abbreviations: AOR, adjusted odds ratio; CI, confidence interval; DBP, diastolic blood pressure; SBP, systolic blood pressure; WHO METS, World Health Organization metabolic equivalents for physical activity.

**TABLE 5:** Binary logistic regression showing predictors of diastolic BP nondipping among the participants.

Predictor	AOR (exp *B*)	95% CI	*p* value	Direction
Income ₦70,000–₦300,000	7.91	0.88–70.80	0.064	Increased risk
Income ₦301,000–₦500,000	9.98	1.24–80.06	0.03	Increased risk
Tertiary education	3.7	1.35–10.10	0.011	Increased risk
Meets WHO METS (≥ 600 METS)	3.97	1.57–10.08	0.004	Increased risk
Waist‐to‐hip ratio	1667.81	3.09–901.435	0.021	Increased risk
Abnormal 24‐h SBP (≥ 130 mmHg)	6.35	1.25–32.31	0.026	Increased risk
Abnormal 24‐h DBP (≥ 80 mmHg)	0.09	0.02–0.55	0.008	Protective

*Note:* *p* values < 0.05 indicate statistical significance.

Abbreviations: AOR, adjusted odds ratio; CI, confidence interval; DBP, diastolic blood pressure; SBP, systolic blood pressure; WHO METS, World Health Organization metabolic equivalents for physical activity.

People with higher monthly incomes (₦70,000–₦300,000 and ₦301,000–₦500,000) were highly likely to exhibit a nondipping pattern for both systolic (AOR = 104.0 and 96.5, respectively; *p* < 0.001) and diastolic BP (AOR = 7.91 and 9.98, respectively; *p* < 0.05) to a very great extent. Besides income, the waist–hip ratio, indicating central adiposity, was another model that could mainly lead to a nondipping pattern (systolic: AOR = 1.95 × 10^4^; diastolic: AOR = 1.67 × 10^3^; *p* < 0.05).

Elevated 24‐h systolic BP (≥ 130 mmHg) was linked with a greater probability of nondipping in both models (AOR range: 6.35–7.63; *p* < 0.05), whereas abnormal 24‐h diastolic BP (≥ 80 mmHg) seemed to be a protective factor (AOR = 0.09–0.84; *p* < 0.05).

In the case of the model for systolic, following the WHO physical activity guidelines (AOR = 0.23; *p* = 0.010), elevated office diastolic BP (AOR = 0.15; *p* = 0.013) and male sex (AOR = 0.40; *p* = 0.044) were the factors that led to a lower probability of nondipping. On the contrary, in a diastolic model, physical activity (AOR = 3.97, *p* = 0.004) and a higher educational level (AOR = 3.70, *p* = 0.011) increased the likelihood of nondipping.

With the present data, it is allowable to state that socioeconomic and anthropometric factors influence nondipping patterns of both systolic and diastolic BP.

## 4. Discussion

This community‐based study offers some of the most detailed insights to date into the prevalence and circadian patterns of abnormal ambulatory BP among Nigerian adults. The results show a high prevalence of nondipping (49.9% and 38.6% for systolic and diastolic, respectively) and reverse dipping (31.5% and 17.3% for systolic and diastolic, respectively) BP profiles, indicating an increased cardiovascular risk in the population. These abnormalities are closely linked to socioeconomic and anthropometric factors, notably higher income and central adiposity, and are influenced by lifestyle choices, including physical activity. Overall, these findings underscore the ongoing epidemiological transition in sub‐Saharan Africa and the need for hypertension management strategies tailored to the local environment.

### 4.1. Lifestyle and Sociodemographic Correlates

The mean age of participants was 48.6 years, which corresponds to the age at which hypertension rates tend to rise significantly, consistent with both national and regional data [1, 5, 21, 22]. Despite 88% of participants having a high level of education, common modifiable risk factors remained evident. Tobacco use was low (< 3%), reflecting some success of public health efforts [23–25], yet alcohol consumption (65%) and high salt intake (around 75%) were widespread. These behaviours indicate dietary shifts in urban settings, characterised by increased intake of processed foods and those high in sodium. Similar trends have been reported in studies from Ghana and South Africa, where urbanisation and changes in food environments have amplified the impact of hypertension and obesity [1, 26, 27]. Although 78.6% of participants adhered to the World Health Organization’s physical activity guidelines, issues such as excessive caloric intake and central adiposity persisted. This indicates that physical activity alone may be insufficient to mitigate dietary risks. This phenomenon exemplifies the complex interaction between energy expenditure and intake during the nutrition transition, wherein traditional activity patterns often persist while dietary practices increasingly promote obesogenic and cardiometabolic risk environments. These behavioural patterns are crucial to understanding the physiological outcomes observed through ambulatory monitoring.

### 4.2. Circadian Patterns of Ambulatory BP

The 24‐h BP profile showed a generally normal diurnal rhythm, with a morning surge, stable daytime levels and a decline at night. However, the degree of nocturnal dipping was significantly reduced, with considerable variation between individuals. Only 22.2% and 36.9% of participants displayed normal systolic and diastolic dipping, respectively, both well below the 60%–70% seen in Western cohorts, such as PIUMA [28] and IDACO [29]. Nevertheless, our findings align closely with observations from both African and African‐ancestry populations. The African American Study of Kidney Disease and Hypertension (AASK) [30] reported nondipping rates of about 76% among African Americans, while the CREOLE study [14] similarly observed a high prevalence of nondipping among hypertensive adults from sub‐Saharan Africa. Similar African studies report comparable rates, with nondipping prevalence often exceeding 50% [13, 31]. The consistency of these findings across geographically diverse populations of African descent suggests that a diminished nocturnal BP decline may constitute a common phenotype rather than an isolated abnormality within these groups [31, 32]. Possible reasons include increased sodium retention, autonomic imbalance, reduced night‐time vagal activity and sleep‐disordered breathing, all exacerbated by obesity and psychosocial stress. Additionally, the frequent occurrence of nondipping BP in populations of African descent suggests that genetic or ancestral biological factors may influence circadian regulation of BP. However, current evidence does not definitively confirm these causal links. Future research should include genomic studies, biomarkers of autonomic function, sleep evaluations and detailed assessments of environmental exposures to better understand these mechanisms.

These results also challenge the universal use of traditional dipping thresholds (10%–20%) and highlight the need for ethnicity‐sensitive ABPM reference ranges for African populations. If nondipping is considerably more common among African populations, it remains unclear whether these thresholds provide optimal cardiovascular risk stratification in this demographic. Future longitudinal studies that correlate ambulatory BP patterns with definitive cardiovascular and renal outcomes are required to ascertain whether population‐specific or ethnicity‐sensitive dipping thresholds may more accurately predict risk. Such research would establish an evidence base to refine the interpretation of ambulatory BP and enhance cardiovascular risk assessment among African populations.

Nondipping and reverse dipping have consistently been associated with adverse cardiovascular and renal outcomes, such as left ventricular hypertrophy, microalbuminuria, progression of chronic kidney disease, stroke and increased cardiovascular mortality. The high prevalence of these patterns in this study highlights the importance of ABPM for the early detection of high‐risk individuals who might otherwise go unnoticed with standard office BP checks [33, 34].

### 4.3. Predictors of Nondipping and Abnormal BP Profiles

The regression models confirmed that central obesity, higher income and elevated 24‐h systolic BP independently predict nondipping status. These findings suggest that socioeconomic progress in semiurban African settings may paradoxically increase cardiovascular risk through dietary habits and occupational factors that promote sedentary lifestyles and central fat accumulation. In contrast, male gender, higher office diastolic BP and adherence to WHO physical activity recommendations appeared to protect against systolic nondipping. Interestingly, the effect of physical activity differed between systolic and diastolic dipping patterns, protective for systolic but associated with increased odds of diastolic nondipping. This paradox may reflect timing effects, where evening exertion delays autonomic recovery and diminishes nocturnal BP decline. Such chronobiological interactions deserve further investigation. The influence of waist–hip ratio in both models underscores the crucial role of central adiposity in vascular and autonomic dysfunction. This supports previous reports identifying waist–hip ratio and BMI as stronger predictors of nocturnal BP abnormalities [35, 36]. Overall, these findings reveal a complex interaction between socioeconomic status, adiposity and behavioural factors in determining circadian BP regulation.

### 4.4. Age and Gender Variations

BP steadily increased with age across all measurement periods, indicating the natural stiffening of the arteries and decreased compliance due to chronic vascular remodelling. Older adults had higher pulse pressures and greater nocturnal systolic loads, aligning with observations from other African populations and global ageing trends [37, 38]. Gender differences were notable, with women showing higher night‐time systolic BP and greater variability, especially postmenopause, likely due to declining oestrogen and increased salt sensitivity. This pattern aligns with studies from South Africa and Kenya and underscores the higher cardiovascular risk among postmenopausal women [37, 39, 40]. In contrast, men displayed lower nocturnal heart rates and more pronounced systolic dipping at younger ages. These sex‐specific patterns highlight the need for gender‐tailored hypertension screening and treatment approaches.

### 4.5. Limitations of the Study

This study has limitations. Its cross‐sectional design prevents causal conclusions about sociodemographic, anthropometric, lifestyle and BP factors; only associations can be inferred. ABPM was measured once every 24 h, and daily factors such as activity and sleep affect the readings. Multiple assessments could improve accuracy. Self‐reported data on physical activity, diet, alcohol and smoking are susceptible to bias, despite standardised questionnaires. Although participants documented their sleep and wake times during ABPM, comprehensive information on occupational schedules, shift work, night shifts, sleep duration, chronotype, sleep quality and sleep disorders was not systematically collected. These variables are recognised to impact circadian BP regulation. The study was conducted in a single semiurban community in south‐western Nigeria. Although a multistage sampling technique was used to enhance representativeness, caution is warranted when extrapolating the findings to other Nigerian populations with different ethnic, cultural, socioeconomic or environmental characteristics.

Despite adjusting for many variables, unmeasured factors like diet, sleep disorders, stress, genetics and autonomic dysfunction might confound results. The ambulatory BP thresholds and dipping classifications used here follow international guidelines, which were mainly derived from non‐African populations. Outcome‐based reference values specific to sub‐Saharan Africans are lacking. Future longitudinal studies with cardiovascular outcomes, repeated ABPM measurements, sleep assessments and genomic research are needed to determine whether population‐specific thresholds can better predict cardiovascular risk in Africans.

Despite these limitations, the study exhibits notable strengths, including its community‐based design, utilisation of validated ABPM, standardised data collection protocols and an extensive assessment of ambulatory BP profiles and circadian rhythms in a sizable African cohort. These attributes offer significant insights into ambulatory BP traits in a context where such data are limited.

### 4.6. Clinical and Public Health Implications

The high prevalence of abnormal ABPM profiles—nearly 40% with elevated 24‐h BP and over 65% with abnormal nocturnal diastolic load—highlights the inadequacy of office BP measurements alone for diagnosing hypertension. These figures surpass those from Western populations but align with other African studies [14, 38, 40, 41], reinforcing the continent‐wide trend towards nocturnal hypertension and nondipping dominance. From a clinical perspective, ABPM offers essential insights into masked and nocturnal hypertension, supporting accurate risk assessment and customised treatment. In the Nigerian setting, routine ABPM could enhance the detection of high‐risk phenotypes often overlooked in outpatient clinics. Public health strategies must therefore extend beyond medication alone to tackle the underlying behavioural and socioeconomic factors that disrupt BP rhythms—particularly obesity, excessive salt intake and sedentary lifestyles. These results also have implications for cardiovascular risk models, which may underestimate the true burden if nocturnal and variability components are ignored. Therefore, integrating ABPM into hypertension guidelines and primary care algorithms should be a priority in sub‐Saharan Africa.

### 4.7. Implications for Practice and Policy

Routine integration of 24‐h ABPM into the diagnosis of hypertension in Nigeria and similar areas could significantly improve accuracy, reduce therapeutic inertia and support personalised treatment, including chronotherapy. Health systems should prioritise waist–hip ratio screening as an accessible cardiovascular risk marker and include lifestyle advice in routine care. Strategies targeting the population should include culturally sensitive salt‐reduction programmes, public awareness of the timing of physical activity and interventions for central obesity. Increasing health insurance coverage and ensuring access to validated ABPM devices are essential for widespread adoption. Adding ABPM to community hypertension programmes has the potential to transform early detection and prevention efforts throughout the region.

In summary, this research shows that abnormal circadian BP patterns, especially nondipping and reverse dipping, are prevalent among Nigerian adults and are influenced by socioeconomic, body composition and lifestyle factors. Central obesity and higher 24‐h systolic BP are primary contributors to these irregularities, whereas physical activity and male sex confer some protective effects. These insights highlight the importance of establishing population‐specific ABPM reference values, implementing targeted prevention strategies and promoting broader use of ambulatory monitoring in primary healthcare. Addressing the fundamental lifestyle and socioeconomic factors behind nondipping is essential to lessen the rising impact of hypertension‐related cardiovascular diseases in sub‐Saharan Africa.

## Funding

No funding was received for this manuscript.

## Conflicts of Interest

The authors declare no conflicts of interest.

## Data Availability

The data that support the findings of this study are available from the corresponding author upon reasonable request.
